# Identification and functional analysis of rare *HECTD1* missense variants in human neural tube defects

**DOI:** 10.1007/s00439-024-02647-4

**Published:** 2024-03-07

**Authors:** Elias Oxman, Huili Li, Hong-Yan Wang, Irene E. Zohn

**Affiliations:** 1 Center for Genetic Medicine Research, Children’s Research Institute, Children’s National Research and Innovation Campus, Children’s National Hospital, Washington, DC 20012 USA; 2 Department of Molecular, Cellular and Developmental Biology, University of Colorado, Boulder, CO 80309 USA; 3 Obstetrics and Gynecology Hospital, Institute of Reproduction and Development, State Key Laboratory of Genetic, Engineering at School of Life Sciences, Fudan University, Shanghai, 200011 China

## Abstract

Neural tube defects (NTDs) are severe malformations of the central nervous system that arise from failure of neural tube closure. HECTD1 is an E3 ubiquitin ligase required for cranial neural tube closure in mouse models. NTDs in the *Hectd1* mutant mouse model are due to the failure of cranial mesenchyme morphogenesis during neural fold elevation. Our earlier research has linked increased extracellular heat shock protein 90 (eHSP90) secretion to aberrant cranial mesenchyme morphogenesis in the Hectd1 model. Furthermore, overexpression of HECTD1 suppresses stress-induced eHSP90 secretion in cell lines. In this study, we report the identification of five rare *HECTD1* missense sequence variants in NTD cases. The variants were found through targeted next-generation sequencing in a Chinese cohort of 352 NTD cases and 224 ethnically matched controls. We present data showing that *HECTD1* is a highly conserved gene, extremely intolerant to loss-of-function mutations and missense changes. To evaluate the functional consequences of NTD-associated missense variants, functional assays in HEK293T cells were performed to examine protein expression and the ability of HECTD1 sequence variants to suppress eHSP90 secretion. One NTD-associated variant (A1084T) had significantly reduced expression in HEK293T cells. All five NTD-associated variants (p.M392V, p.T801I, p.I906V, p.A1084T, and p.P1835L) reduced regulation of eHSP90 secretion by HECTD1, while a putative benign variant (p.P2474L) did not. These findings are the first association of *HECTD1* sequence variation with NTDs in humans.

**Supplementary Information:**

The online version contains supplementary material available at 10.1007/s00439-024-02647-4.

## Introduction

Neural tube defects (NTDs) are severe congenital malformations of the spine and brain. They are among the most common structural birth defects, with a global prevalence of 0.5–10 per 1000 live births (Greene and Copp 2014). The occurrence of NTDs in various populations is affected by genetics, geography, and the maternal diet (Li et al. 2006; Liu et al. 2016). For instance, in Northern China’s Shanxi Province in 2003, the frequency of NTDs was 138.7 per 10,000 births, tenfold higher than in the United States and Europe (Li et al. 2006; Morris and Wald 2007; Williams et al. 2015). The diet in this region was shown to be deficient in folate, and the incidence of NTDs in this region was significantly reduced following a campaign to provide folic acid supplementation (Liu et al. 2016; Meng et al. 2015). NTDs arise in the first trimester of pregnancy from defects in neurulation, a process where the neural plate transforms into a tube to form the central nervous system (Wallingford et al. 2013). NTDs can occur at different axial levels, resulting in anencephaly in the head, spina bifida in the spine, or craniorachischisis with the complete failure of neural tube closure along the entire neural axis. Anencephaly and craniorachischisis are fatal, whereas spina bifida can result in significant disability.

The causes of NTDs are polygenic and multifactorial and, estimates suggest that ~70% of NTDs have a significant genetic component (Copp et al. 2015; Jorde et al. 1983; Lupo et al. 2017). For instance, the relative risk among first-degree relatives increases to 3% (Jorde et al. 1983), and NTDs are more frequent in some genetic syndromes and chromosomal anomalies (Copp et al. 2015; Lupo et al. 2017; Toriello and Higgins 1983). Genome-wide sequencing projects indicate that rare and novel variants in NTD candidate genes may contribute to NTDs in an oligogenic fashion (Chen et al. 2018c; Ishida et al. 2018). For example, screening a cohort of 90 cases with cranial NTDs from northeast England between 1992 and 2011 with a targeted exome sequencing panel of 191 genes identified 397 rare variants (Ishida et al. 2018). On average, NTD cases had nine rare/novel variants, three of which were predicted to be damaging. In contrast, case–control samples had an average of two novel/rare variants, with 1.5 predicted to be damaging (Ishida et al. 2018). In analyzing whole-genome sequencing (WGS) data from three different NTD cohorts (Han Chinese, Caucasian USA, and Middle Eastern/Qatar) with various NTD types, researchers found a higher occurrence of singleton loss-of-function (SLoFVs) variants among NTD cases than controls (Chen et al. 2018c). SLoFVs were defined as variants that appear only once in the 1000 genome project. Based on these findings, the authors suggest that the number of SLoFVs is a stable and reliable genomic indicator of NTD risk in humans, with nine SLoFVs a genomic threshold for NTD risk (Chen et al. 2018c).

HECTD1 is a HECT domain E3 ubiquitin ligase that targets proteins for degradation or alters their function. We initially identified the mouse *Hectd1* gene in an ENU mutagenesis screen to identify genes required for neural tube closure (Kasarskis et al. 1998; Zohn et al. 2007). This novel ENU-induced *Hectd1* mutant mouse model (*openmind*, *opm*) exhibited fully penetrant exencephaly in homozygous *Hectd1*^*opm/opm*^ embryos and incomplete penetrance in heterozygotes (Zohn et al. 2007). Depending on the mutation, 5 to 20% of heterozygous *Hectd1* mutant mouse embryos showed exencephaly (D’Alonzo et al. 2019; Zohn et al. 2007). Our study of the developmental mechanism leading to exencephaly in the *Hectd1* mouse model revealed that the defect arises from the abnormal morphogenesis of the cranial mesenchyme (Sarkar and Zohn 2012; Zohn et al. 2007), a process required to elevate the cranial neural folds (Morris-Wiman and Brinkley 1990a, b, c; Morriss and Solursh 1978a, b; Zohn and Sarkar 2012). Analysis of the pathways regulated by HECTD1 in the cranial mesenchyme implicated increased secretion of extracellular Hsp90 (eHsp90) as a likely cause of the cranial mesenchyme and neural tube closure defects in *Hectd1* mutant embryos (Sarkar and Zohn 2012). Our research demonstrated that eHSP90 stimulates the migration of cranial mesenchyme cells, interfering with normal cranial mesenchyme morphogenesis and neural fold elevation. Likewise, the expression of HECTD1 in HEK293T cells suppresses the stressed-induced secretion of eHSP90 (Sarkar and Zohn 2012).

The present study identifies five case-specific missense variants in the *HECTD1* gene from a Chinese NTD cohort. Based on our prior knowledge that NTDs in the *Hectd1* mutant mouse model are due to elevated secretion of eHSP90 stimulating cranial mesenchyme migration (Sarkar and Zohn 2012), we utilize this as an assay to functionally test the impact of missense variants on HECTD1 function. Our findings indicate that all variants associated with NTDs showed activity loss, whereas a putative benign *HECTD1* variant did not. These data suggest sequence variation in *HECTD1* may contribute to human NTDs.

## Materials and methods

### Human subjects and targeted next-generation sequencing of *HECTD1*

The subjects of this study were previously described (Chen et al. 2018a, b; Lei et al. 2019; Qiao et al. 2016; Shi et al. 2018; Ye et al. 2020). 352 NTD samples were collected from aborted fetuses and children with spina bifida from the 1990s to the 2010s in 5 Chinese provinces. Tissue samples were collected from 309 aborted fetuses (23.4 ± 6.7 weeks) with craniorachischisis, encephalocele, anencephaly, exencephaly, spina bifida, or more than one type of NTD. Whole blood samples were obtained from 43 children (6.4 ± 4.6 years) with spina bifida. 224 control samples (58.9% female, 40.6% male, 0.5% unknown) were ethnically and gender-matched unrelated healthy volunteers recruited from Shanxi (aborted healthy fetuses) and Shanghai (blood samples from healthy first-year college students) in China. Protocols were reviewed and approved by the Ethics Committee of the School of Life Sciences, Fudan University. The coding region of *HECTD1* was sequenced by targeted next-generation sequencing as previously described (Qiao et al. 2016). Variants were assessed for predictive deleteriousness using SIFT (http://sift.jcvi.org/) and Mutation Taster2 (http://www.mutationtaster.org/). *HECTD1* variants analyzed in this study were absent in controls, the 1000 genome project (http://www.1000genomes.org), or extremely rare in the Genome Aggregation Database (gnomAD; http://gnomad.broadinstitute.org/).

### Cell culture and DNA transfection

**Fig. 1:**
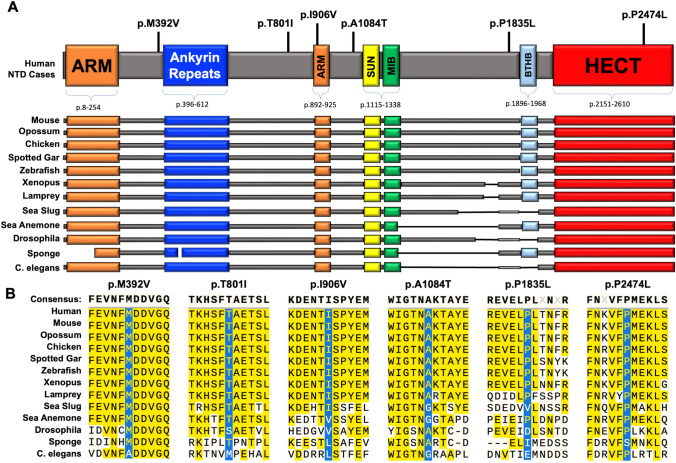
NTD-associated missense variants in *HECTD1*. **a** Domain structure of HECTD1 showing armadillo (ARM, orange) and ankyrin repeats (ANK, blue), Sad1/UNC (SUN, yellow), mind-bomb domain (MIB, green), Basic Tilted Helix Bundle (BTHB, light blue), and Homologous to the E6-AP Carboxyl Terminus domain (HECT, red) domains. The position of human NTD-associated missense variants and the putative benign p.P2474L variant are indicated. **b** The multiple-sequence protein alignment was done using MUSCLE. The amino acid sequences adjacent to the NTD-associated missense variants are shown in the various HECTD1 orthologues. Sequences were obtained through Blastp. The accession numbers, % query coverage, and *E*-value are listed in Table 2. The amino acids altered by missense variants are absolutely conserved among vertebrates, with some conservation into invertebrate taxonomic groups

HEK293T cells from American Type Culture Collection (CRL-3216) were maintained in high glucose DMEM media (Gibco 11995065) supplemented with 10% Fetal bovine serum and Penicillin–Streptomycin and grown in a 5% CO_2_ humidified incubator at 37 °C. *pCMV-HA-Hectd1, pCMV-HA-Hectd1(C2579G),* and *pCMV-Hsp90a-Myc* constructs were previously described (Sarkar and Zohn 2012). The p.C2579G variant is an engineered construct where the active site cysteine in the HECT domain is mutated, abolishing the ubiquitin ligase activity of HECTD1. Aligent QuikChange II XL Site-Directed Mutagenesis (Aligent 200521) was used to introduce missense mutations confirmed by Sanger sequencing. *CD63-pEGFP C2* (gift from Paul Luzio, Addgene plasmid 62964; https://www.addgene.org/62964/; RRID: Addgene_62964) was used as a transfection control in the immunofluorescence assay (Fig. 4). *pRK5-HA-GFP* (HA-GFP gift from Carol Mercer, Addgene plasmid 137763; http://n2t.net/addgene:137763; RRID: Addgene_137763; Stefely et al. 2020) was used as a loading control in the western blot analysis (Fig. 3). Plasmids were prepared using the Qiagen Mini-Prep Kit (Qiagen 27104) and cut with PvuII-HF restriction enzyme (NEB R3151S) to confirm quality and concentration. HEK293T cells were transfected using Lipofectamine 3000 Reagent (L3000008) according to the manufacturer’s instructions with either 730 ng wild-type or mutant *pCMV-HA-Hectd1*, 400 ng *pCMV-Hsp90a-Myc*, and 50 ng *CD63-pEGFP C2* (immunofluorescence assay, Fig. 4) or 1 ug *pCMV-HA-Hectd1* and 50 ng *pRK5-HA-GFP* (western blot analysis Fig. 3).

### Western blot analysis

Transfected cells were expanded to a six-well plate 24 h after transfection and lysed 48 h post-transfection. Lysis was performed using IP Lysis Buffer (Pierce, 87787) with diluted 100× Halt Protease Inhibitor Cocktail (78429). Plates were rocked at 4 °C for 10 min, scraped, and debris was separated by centrifuging 13,000×*g* for 10 min at 4 °C. The supernatant was collected, and concentration was quantified using a Pierce BCA Protein assay kit (23225) or Coomassie Protein Assay Reagent (Thermo Scientific 1856209). Lysate was prepared in NuPAGE 4× Sample Buffer (NP0007), NuPAGE Reducing Agent 10× (NP0004), heated at 70 °C for 10 min, and resolved on 10-well NuPAGE 3–8%, Tris–acetate Mini Protein Gel (EA0375) with NuPAGE Tris–Acetate SDS Running Buffer (LA0041) to detect HA-HECTD1 or 10-well NuPAGE 4–12%, Bis–Tris Mini Protein Gel (NP0321) with the NuPAGE MES SDS Running Buffer (NP0002) to detect HA-GFP run at 150 V for 1.5 h. Protein was transferred using a low fluorescence PVDF Transfer Membrane (22860) at 20 V for 1.5 h. Transfer buffer was supplemented with NuPAGE Transfer Buffer 20x (NP0006), 10% Methanol, and NuPAGE Antioxidant (NP0005). Membranes were blocked with LiCOR Intercept Blocking Buffer (927–60,001) for 1 h at room temperature. Primary antibodies were incubated overnight at 4 °C at the following dilutions: HA-HECTD1 and HA-GFP were detected using anti-HA.11 (Clone 16B12, BioLegend) diluted 1:750 and 1:1000, respectively, in Intercept Antibody Dilutant (927–65,001). Secondary antibody IRDye 800CW donkey anti-mouse (92,632,212; 1:15,000 dilution) was incubated for 1 h at room temperature. Blots were imaged using the LiCor Odyssey CLX imaging system and LI-COR software. Exported image files were processed to 800 channel only, inverted LUT, and integrated density was measured over 3 established Regions of Interest (ROIs). Relative expression was measured through averaged integrated density values and calculated by normalizing HA-HECTD1 expression to transfection control HA-GFP. Statistical analysis was performed using Prism GraphPad 9, and the significance was determined using the two-tailed Student’s *t* test with a Bonferroni corrected *p*-value for multiple testing of *p* < 0.006. Four replicates were used in the western analysis; p.A1084T is reported in triplicate due to a technical error. HA-HECTD1 and p.P2474L were assayed using the same method independently for three replicates.

### eHSP90a secretion assay

Transfected cells were passaged 24 h after initial transfection to a 6-well plate with a sterilized coverslip in the well. 24 h after passaging cells, respective wells were treated with 10 micromolar *N*-acetyl-*N*-Acetyl-Leu-Leu-Nle-CHO (ALLN, BML-P120) for 1 h. Cells were washed with 1× PBS with calcium and magnesium (Gibco 14040141) and fixed for 20 min with 4% Paraformaldehyde. Blocking buffer for primary antibodies and washes were formulated with 1× PBS (Sigma, P3813) and 1% heat-inactivated goat serum (16210064). Primary incubations were done for 1 h at room temperature with Myc-Tag (71D10) Rabbit mAb (1:200 Cell Signal, 2278), and secondary incubations for 1 h at room temperature in the dark with Hoechst 33342 Solution (1:500, 62249) and Alexa-Fluor Anti-Rabbit 555 (1:250, A-21428). Final washes were performed with a blocking buffer with 0.1% Triton—X100 to reduce excess signal. Coverslips were mounted using Fluoromount-G™ Mounting Medium (00-4958-02). Slides were imaged using the Leica TCS8 Confocal Microscope with a 63×-magnification oil immersion objective (NA = 1.4). Image acquisition was made blind by randomization of slides. Images were acquired on detection of CD63-GFP transfection control, and then parameters were adjusted for detection of extracellular Hsp90a or lack thereof. The maximum projection of the Z stack was applied to images in ImageJ. Images of cells acquired during blind acquisition had to exhibit standard nuclei shape and health to ensure intact plasma membrane. Final images were processed using a coding key and scored by five independent reviewers to reach a consensus on categorizing myc-eHSP90 staining as “No secretion,” “Moderate secretion,” or “Excessive secretion.” After the scoring was performed, slides were unblinded for statistical analysis. Chi-squared tests were performed to test significance with a Bonferroni corrected *p*-value for multiple testing of *p* < 0.0055. ALLN treatment conditions were assessed independently. Three replicates were performed, consisting of 5 frames per replicate for 15 frames per condition. HECTD1 and p.C2579G were assayed with 20 replicates after including p.P2474L in a separate assay.

### Multiple sequence alignment of HECTD1 protein sequences

To obtain the sequences for the graphical and multiple sequence alignments of HECTD1, a series of blastp queries were performed strategically, moving through lower-order organisms. The subject sequence was obtained from UniProt as the canonical HECTD1 *Homo sapiens* protein sequence (Q9ULT8). Standard laboratory animal models were first queried (*M. musculus, G. gallus, X. laevis, D. rerio, D. melanogaster, C. elegans*). Additional queries were performed: opossums (taxid: 9265), bony fish (taxid: 7898), lampreys (taxid: 7745), cnidaria (taxid: 6073), sponges (taxid: 6040), and anasdipea (taxid: 6497). Named proteins and X1 isoforms (where applicable) were chosen as sequences for multiple sequence alignment. blastp (https://blast.ncbi.nlm.nih.gov/Blast.cgi?PAGE=Proteins) queries were performed using default parameters. Sequences were aligned using MUSCLE with the UPGMA clustering method, 1.20 hydrophobicity multiplier, −2.90 gap open, and Lambda of 24. Alignment was visualized utilizing SnapGene.

### AlphaFold rendering of human NTD-associated variants

AlphaFold v2.3.0 (Jumper et al. 2021) renderings were performed on the Children’s National Research Institute High-Performance Computing cluster. The human canonical sequence (Uniprot Q9ULT8) was edited into multiple FASTA files with missense variants. Each missense variant FASTA protein sequence was run using the monomer_ptm preset and 9/8/2022 max template date. Each variant’s zero-ranked PDB (protein database format) output was imported into YASARA (yet another scientific artificial reality application) and superimposed on the zero-ranked wild-type PDB output. Residues of interest were transformed to the ball and stick view for structural and confidence scores with the predicted local distance difference test (pLDDT). The pLDDT score is a measure of confidence determined by the tolerated distance between a specific residue and others within the structure on a scale of 0–100, where a higher score indicates a greater confidence (Mariani et al. 2013). AlphaFold has been a recent subject of scrutiny to improve in silico predictions for pathogenicity. pLDDT scores reveal metrics for determining the tolerance of mutations and their structural impact. For instance, prior studies have found that regions with low pLDDT confidence scores demonstrate lower incidences of VUS and pathogenic variants in established disease genes (Schmidt et al. 2023). The pLDDT scores are included for wild type and missense residues and are in the range of 70–90, indicating they were modeled well by the algorithm but not with the highest accuracy (>90); this still supports the functional importance of the implicated areas. However, the lack of variance in pLDDT scores between wild type and missense variant models indicates that the missense mutation alone does not alter AlphaFold’s ability to render the localized region confidently.

## Results

### Identification of *HECTD1* missense variants in human NTD cases

Targeted next-generation sequencing of *HECTD1* in a well-characterized NTD cohort of 352 NTD cases and 224 ethnically and gender-matched controls identified 5 rare (minor allele frequency <0.01) heterozygous missense variants (Table 1 and Fig. 1a). None of the variants were found in the ethnically and gender-matched controls. Evaluation of these variants across publicly available databases indicated that variants are either exceedingly rare or unique. The c. 1174A>G (p. M392V), c. 2716A>G (p. I906V), c. 2716A>G (p. A1084T) and c.5504C>T (p.P1835L) were rare, with gnomAD frequencies of 2.09e−6 (2.723e−6 in the European non-Finnish but not reported in the East Asian ancestry group), 1.86e−5 (2.233e−5 among the East Asian ancestry group), 7.97e−6 (1.389e−4 among the East Asian ancestry group), and 6.84e−7 (8.996e−7 in the European non-Finnish but not reported in the East Asian ancestry group), respectively. One variant, c.2402C>T (p.T801I), was novel and not found in the gnomAD dataset or the ethnically matched control cohort. All but one variant (p. I906V) is predicted to be damaging/deleterious by PolyPhen-2 and SIFT variant effect prediction tools (Adzhubei et al. 2010; Ng and Henikoff 2003). As a control, this study also includes a reported benign (ClinVar) variant, p.P2474L (rs111683057). This variant has a gnomAD frequency of 1.1e−3, primarily in the African/African American ancestry group (2.043e−2), with 9 homozygous individuals reported.

**Table 1:** Summary of *HECTD1* variants and clinical information reported in this study

Sample ID	rsID	Nucleotide change	Amino acid change	PolyPhen2	SIFT	Morphological phenotype	Age	Sex	gnomAD Frequency (All)	gnomAD Frequency (East Asian)
D19	N/A	c.1174A>G	p.M392V	0.872 Damaging	0.01 Deleterious	Lumbar sacral spina bifida aperta, right lung malformation	25W	F	0.00	0.00
D121	N/A	c.2402C>T	p.T801I	0.838 Possibly Damaging	0 Deleterious	Anencephaly, meningoencephalocele	20W	F	0.00	0.00
D2	rs757106907	c.2716A>G	p.I906V	0.025 Benign	0.14 Tolerated	Craniorachischisis	19W	M	1.86e−5	2.233e−5
QQHE84	rs762560439	c.3250G>A	p.A1084T	0.985 Damaging	0 Deleterious	Encephalocele	ND	ND	7.97e−6	1.389e−4
D115	N/A	c.5504C>T	p.P1835L	0.608 Possibly Damaging	0 Deleterious	Craniorachischisis; right lung lobe malformation; short neck and trunk, thoracic cage deformity, long limbs	20W	F	6.84e−7	0.00
Benign control	rs111683057	c.7421C>T	p.P2474L	0.085 Benign	0 Deleterious	N/A	–	–	1.1e−3	0.00

*N/A *not applicable; *ND *no data

The *HECTD1* missense variants alter conserved amino acids localized within defined and well-conserved functional domains of HECTD1 or amino acids just flanking these domains (Fig. 1a). Our previous results indicate that HECTD1 interacts with HSP90 through the N-terminal armadillo and ankyrin repeats (Sarkar and Zohn 2012). SUN (Sad1-UNC-84 homology) domain and Basic tilted helix bundle (BTHB) domains can also mediate protein–protein interactions (Dilworth et al. 2017; Starr and Fridolfsson 2010). A multiple sequence alignment was performed (Fig. 1b) utilizing MUSCLE (MUltiple Sequence Comparison by Log- Expectation) to align the sequences of selected species (Starr and Fridolfsson 2010). All residues implicated in our missense variants were universally conserved among vertebrates, and several were also preserved in the invertebrate species surveyed.

**Fig. 2:**
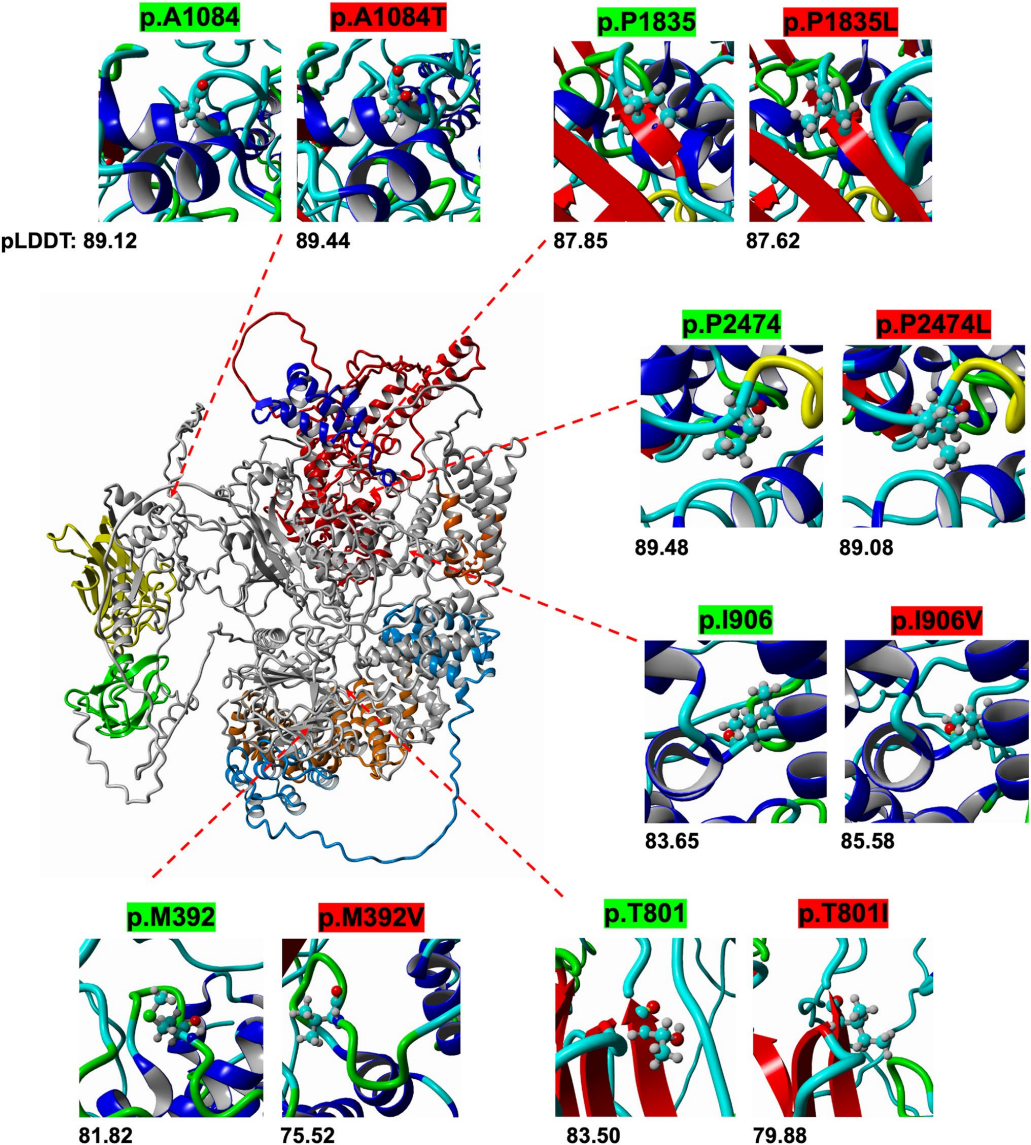
AlphaFold Rendering of HECTD1. Rendering displays a color overlay of defined domains matching the color scheme shown in Fig. 1a. Residues of interest are magnified and illustrated in ball and stick format. The coloration of magnified images depicts the pLDDT scores of respective residues. Models are shown superimposed and aligned based on the highest ranked output

AlphaFold analysis of HECTD1 protein structure suggests missense variants do not result in significant changes to tertiary or secondary structure (Fig. 2). The pLDDT scores do not indicate relevant deviations from the wild-type structure. Differences in pLDDT scores are represented in coloration changes of the b-factor field in the 3D rendering of the PDB output and text output shown underneath each frame (Fig. 2). Significant conformational changes would have been indicative of destabilizing variants.

### *HECTD1* is a highly conserved and constrained gene

Phylogenetic analysis reveals that the amino acid sequence of HECTD1 is evolutionarily constrained. An extensive blastp of the canonical human protein sequence (Uniprot Q9ULT8) against the major animal taxa illustrates this conservation. Figure 1a shows a graphical summary of the MUSCLE alignment (Starr and Fridolfsson 2010), indicating that nearly all domains are conserved in HECTD1 down to invertebrates such as *C. elegans.* Sequence conservation is lowest in the intervening region between the MIB and BTHB domains. Individual blasts of these regions did not yield any significant results for functional purposes or orthologs outside of the respective taxa. Within invertebrates, this region may be novel to each taxa. Table 2 illustrates the findings of the blastp, indicating that all tested organisms have significant query coverage and sequence identity to the canonical human form.

**Table 2:** Blastp results of comparing human HECTD1 against the indicated species for comparative analyses in Fig. 1b

Species (common name)	Accession	Query coverage	*E*-value	Percent identity	Total score
*Mus musculus* (Mouse)	NP_659037.2	100%	0	98.93%	5356
*Gallus gallus* (Chicken)	XP_421227.4	100%	0	95.60%	5177
*Xenopus laevis* (Frog)	XP_018087672.1	96%	0	89.55%	4554
*Danio rerio* (Zebrafish)	NP_001002504.2	100%	0	86.88%	4462
*Drosophila melanogaster* (Fruit Fly)	NP_609369.1	89%	0	53.50%	2481
*Monodelphis domestica* (Opossum)	XP_001364091.1	100%	0	97.82%	5301
*Lepisosteus oculatus* (Spotted Gar)	XP_015205628.1	100%	0	89.90%	4581
*Petromyzon marinus* (Sea Lamprey)	XP_032812070.1	96%	0	77.28%	3923
*Aplysia californica* (Sea Slug)	XP_005090788.1	86%	0	62.66%	2843
*Nematostella vectensis* (Sea anemone)	XP_048585862.1	84%	0	65.77%	2823
*Amphimedon queenslandica* (Sponge)	XP_019857021.1	74%	0	47.81%	1703
*Caenorhabditis elegans* (Roundworm)	NP_001368365.1	79%	0	52.31%	1788

Sequence variation in human *HECTD1* is under notable constraint. The gnomAD database indicates that loss of function variants are significantly underrepresented in the *HECTD1* gene. *HECTD1* loss of function variants have a LoF intolerant (pLI) score of 1 and a LOEUF score of 0.27 (50 observed vs. the expected 238.4 LOF variants). Since haploinsufficient genes often have a high pLI score and a low LOEUF score (Gudmundsson et al. 2022; Lek et al. 2016), these data indicate that *HECTD1* could be a haploinsufficient disease gene. The *HECTD1* gene is also highly constrained for missense variation with a Z score of 6.42 (1911 observed SNVs vs. 2849.1 expected SNVs). Moreover, *HECTD1* has an RVIS (Residual Variation Intolerance Score) of −2.23, ranking among the 1.33% most intolerant to functional genomic changes of all human genes (Petrovski et al. 2013). These metrics suggest that missense variation and loss of function changes in *HECTD1* are poorly tolerated compared to most human genes. Additionally, this high level of constraint could be consistent with sequence variation in *HECTD1* causing disease in heterozygous individuals (Gudmundsson et al. 2022; Lek et al. 2016).

### The p.A1084T variant showed reduced expression in HEK293T cells

**Fig. 3:**
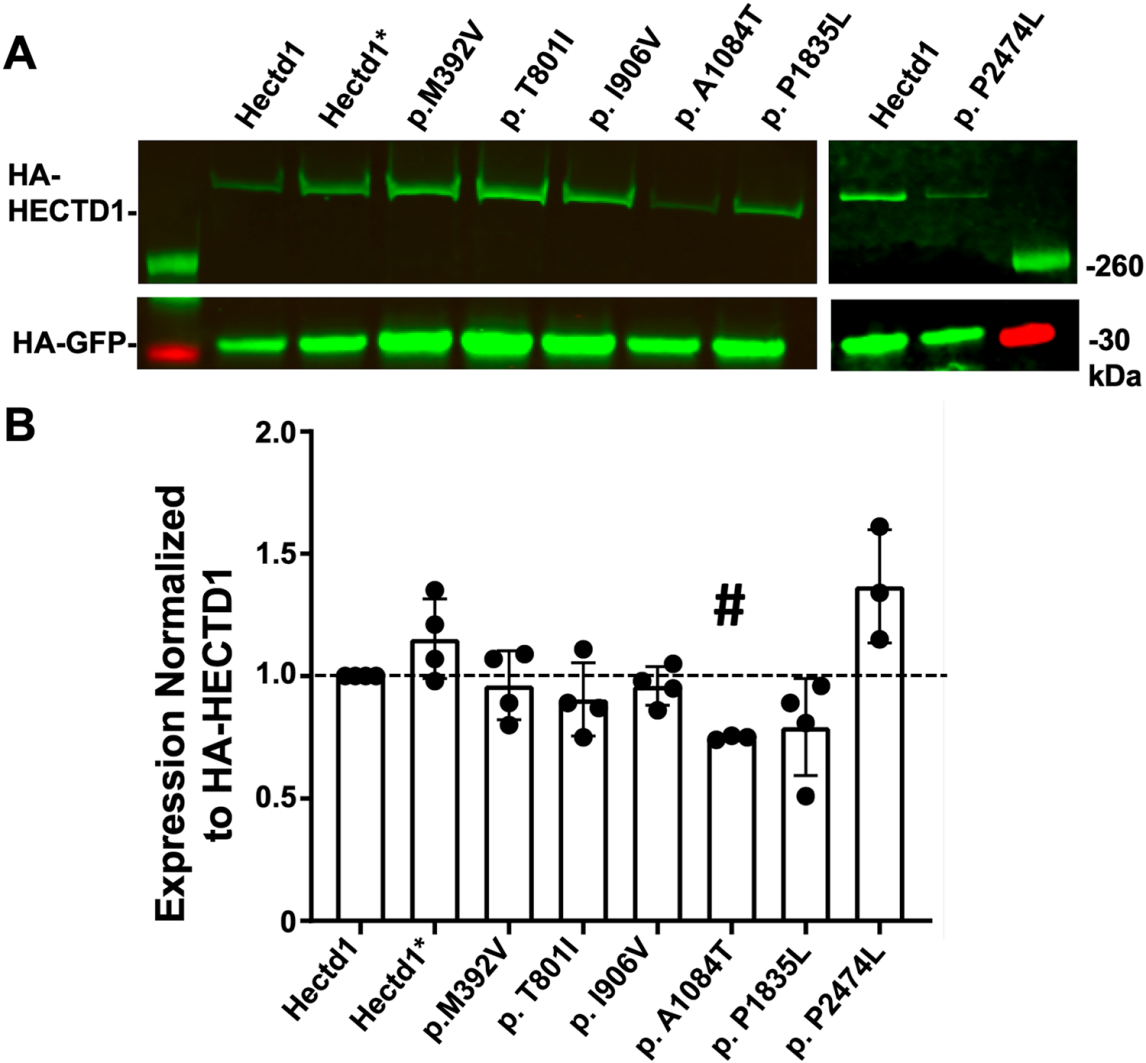
Expression of *HECTD1* missense variants in HEK293T cells. **a** Western blot analysis of *pRK5-HA-Hectd1* expression levels in HEK293T cells co-transfected with *HA-GFP* as a transfection control. **b** Expression was normalized to HA-GFP and then wild type HECTD1 and averaged across four independent experiments. Transfection of the NTD-associated variant, p.A1084T, showed a significantly reduced expression level (two-tailed Student’s *t*-test *p* < 1e−6, *n* = 3, indicated by “#”, with a Bonferroni corrected *p*-value for multiple testing of *p* < 0.006) compared to the Hectd1 wild-type transfected cells. Differences in expression of the other NTD-associated variants (*n* = 4), putative benign p.P2474L (*n* = 3), and the engineered cysteine mutant p.C2579G (Hectd1*) were not significant

First, we tested if missense variants alter the expression levels of HECTD1 in HEK293T cells. Western blot analysis of transiently transfected HEK293T cells shows that the p.A1084T variant exhibited significantly reduced expression in three independent experiments (*p* < 1e−6). In contrast, the expression of the other NTD-associated variants and the benign p.P2474L variant, the engineered loss of function p.C2579G variant (Hectd1*) were not significantly altered (Fig. 3).

### NTD-associated *HECTD1* sequence variants show reduced activity

**Fig. 4:**
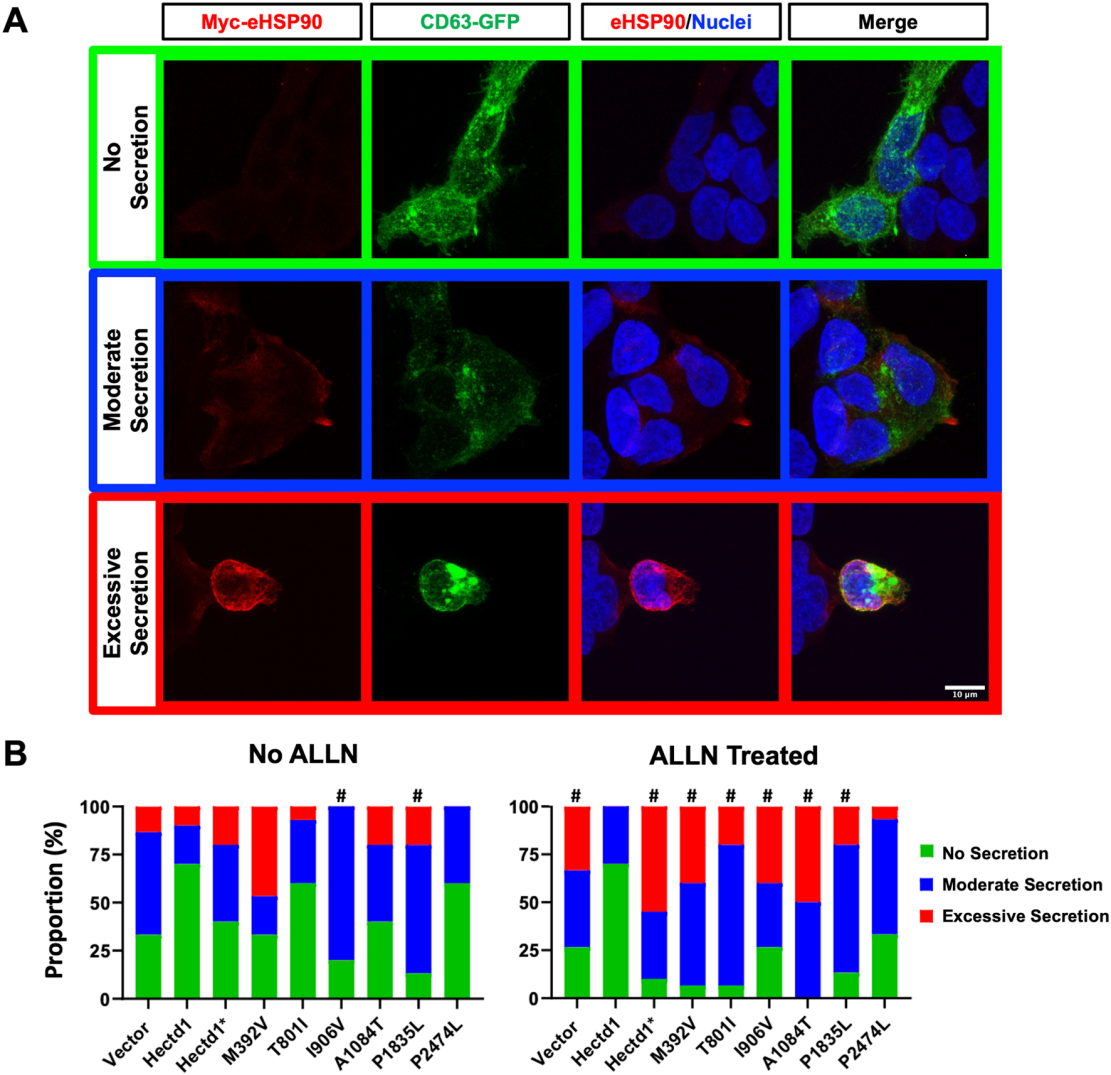
Functional analysis of *HECTD1* missense variants in HEK293T cells. **a** Representative images of activity scored for eHSP90 secretion: No secretion (Green), moderate secretion (Blue), and excessive secretion (Red). Co-transfected CD63-GFP is shown to identify transfected cells. Blocking and incubation of primary antibodies were performed without adding detergent to ensure the detection of extracellular eHSP90. **b** Relative distributions of activity score per frame are shown as a percentage of the total number of frames scored. Three replicates were performed, consisting of five frames per replicate for a total of 15 frames per condition. Each of the five observers scored replicates to reach a consensus on the level of eHSP90 secretion. The Chi^2^ test with a Bonferroni determined significance corrected *p*-value for multiple testing of *p* < 0.0055, comparing each variant to the wild type. See Table 3 for Chi^2^ and *p* values. Without ALLN treatment, the ability of the p.P2474L and p.T801I variants to suppress eHSP90 secretion was not significantly different from wild type HECTD1; the remaining variants showed a significant activity loss. Upon treatment with ALLN, all variants except the benign p.P2474L showed significant activity loss compared to the wild type

Our previous studies demonstrate that increased secretion of extracellular HSP90 (eHSP90) through the exosome pathway in *Hectd1* mutant cells stimulates abnormal migration of the cranial mesenchyme in an explant assay (Sarkar and Zohn 2012). We showed that overexpression of HECTD1 reduces eHSP90 secretion in HEK293T cells in response to stress induced by treating cells with the protease inhibitor ALLN. Conversely, expression of the engineered cysteine mutant Hectd1* (p.C2579G) construct has no activity in this assay, representing an extreme loss of function variant (Sarkar and Zohn 2012). Thus, this assay can functionally test whether NTD-associated *HECTD1* missense variants retain the functionality of preventing eHSP90 secretion, a role related to NTDs. HEK293T cells were transfected with *Myc-Hsp90*, *CD63-GFP*, and the *HA-Hectd1* variant constructs. CD63-GFP was included to identify transfected cells. Immunostaining was done without permeabilization with detergent to detect only extracellular Myc-HSP90. CD63-GFP is detected inside the cell in the endosome compartment, at the cell surface, and in exosomes (Escola et al. 1998). Multiple independent reviewers scored the assay for the presence and intensity of eHSP90 staining on the surface of CD63-GFP-expressing cells. As shown in representative images in Fig. 4a, data were scored as “No secretion” (green), “Moderate secretion” (blue), or “Excessive secretion” (red). Variants p.M392V, p.I906V, and p.P1835L demonstrate activity loss indicated by an increase in the proportion of cells scored with “moderate” secretion of eHSP90 (Fig. 4b, No ALLN). This experiment was repeated in the presence of the protease inhibitor ALLN, which stimulates the secretion of eHSP90 (Sarkar and Zohn 2012). Upon the addition of ALLN, all NTD-associated sequence variants, but not the benign p.P2474L variant, showed reduced activity as measured by an increased proportion of cells scored with “moderate” or “excessive” secretion of eHSP90 compared to the wild type HECTD1.

**Table 3:** Summary of the functional analysis shown in Fig. 4

	No ALLN		ALLN treated	
	Chi^2^	*p* value	Chi^2^	*p* value
Vector	4.98	0.083	10	0.007^#^
Wild type	–	–	–	–
p.C2579G	3.64	0.162	20	0^#^
p.M392V	6.60	0.037	17.2	0^#^
p.T801I	0.834	0.66	14.7	0.001^#^
p.I906V	12.7	0.002^#^	11.2	0.004^#^
p.A1084T	3.15	0.207	20.7	0^#^
p.P1835L	11.3	0.004^#^	12.5	0.002^#^
p.P2474L	2.83	0.243	5.26	0.072

The effect of NTD-associated HECTD1 sequence variants on eHSP90 secretion was determined in HEK293T cells. At baseline (No ALLN) or with ALLN treatment. For significance testing, the Chi^2^ test was used with a Bonferroni corrected threshold *p*-value of *p* < 0.0055 (indicated by “#”) in comparison to Hectd1 wild-type transfected cells

## Discussion

This study identified five case-specific rare missense variants in the *HECTD1* gene in a well-described Chinese NTD cohort (Chen et al. 2018a, b; Lei et al. 2019; Qiao et al. 2016; Shi et al. 2018; Ye et al. 2020). Notably, the missense variants identified in *HECTD1* in NTD cases were not found in the ethnically matched control cohort and are extremely rare in reference population databases. Previous studies demonstrated that *Hetch1* encodes an E3 ubiquitin ligase required for neural tube closure in mouse models (D’Alonzo et al. 2019; Zohn et al. 2007). We now show evidence that *HECTD1* is a highly conserved gene across the animal kingdom. The NTD-associated sequence variants identified in our study alter evolutionarily conserved amino acids within or adjacent to conserved functional domains of the protein. Finally, functional analysis demonstrates missense variants reduced HECTD1 activity, further supporting the possibility of pathogenicity.

Our previous studies indicate that increased eHSP90 secretion results in abnormal morphogenesis of the cranial mesenchyme, disrupting neural fold elevation and leading to NTDs in *Hectd1* mutant mouse embryos (Sarkar and Zohn 2012). Thus, we assessed the ability of HECTD1 to suppress eHSP90 secretion as an assay to determine if NTD-associated sequence variants resulted in loss of HECTD1 activity. NTD-associated *HECTD1* missense variants were expressed in HEK293T cells to determine if they altered protein expression or reduced gene function. All NTD-associated variants tested showed reduced activity in suppressing the secretion of eHSP90. Since the p.A1084T variant was also expressed at significantly lower levels when transfected into HEK293T cells, reduced activity in the eHSP90 secretion assay may be due to decreased expression rather than attenuated activity. Together, these data demonstrate that the sequence changes reduce activity related to the etiology of NTDs, supporting a role for sequence variation in *HECTD1* contributing to NTDs in humans.

Animal studies have identified hundreds of genes involved in forming the neural tube (Harris and Juriloff 2007, 2010; Ishida et al. 2018; Wilde et al. 2014). Searching for rare and novel sequence variants in these NTD candidate genes in NTD cases has been a powerful tool for revealing the genetic causes of human NTDs (Ishida et al. 2018; Wolujewicz and Ross 2019). Rare variants in genes under constraint are particularly interesting in rare disease research due to the anticipated stronger effects (Gudmundsson et al. 2022). We analyzed the Genome Aggregation Database (gnomAD v4.0.0) (Chen et al. 2022; Karczewski et al. 2020) to determine which NTD candidate genes are tolerant and intolerant of missense variation (Supplemental Table 1). Many NTD candidate genes are relatively tolerant of missense variation. For instance, multiple *VANGL1* missense variants have been identified in NTD cases, and the pathogenesis of many of these variants has been validated in numerous studies (Bartsch et al. 2012; Cai et al. 2014; Cheng et al. 2021; De Marco et al. 2014; Doudney et al. 2005; Fatima et al. 2022; Humphries et al. 2020; Iliescu et al. 2014, 2011; Kibar et al. 2007, 2009; Merello et al. 2015; Reynolds et al. 2010; Tian et al. 2020a, b; Wang et al. 2018). *VANGL1* tolerates missense but not loss of function (LOF) variants with a missense Z score of 0.59 but a loss intolerance probability (pLI) scores close to 1 (0.91) and a LOF observed/expected upper bound fraction (LOEUF) score of 0.53. This is consistent with the proposed digenic and multigenic origins of NTDs involving sequence variation in *VANGL* genes (Juriloff and Harris 2012; Torban et al. 2008; Wang et al. 2018; Zohn 2012; Zohn and Sarkar 2008). In contrast, only a handful of NTD candidate genes, including *HECTD1,* exhibit substantial selection against missense variation (Supplemental Table 1). Since causal LOF variants for Mendelian and severe complex diseases are enriched in ‘mutation intolerant’ genes (Agarwal et al. 2023), the strong selection against LOF and missense variants in *HECTD1* provides further support to the idea that deleterious sequence variation in the *HECTD1* gene would significantly affect embryonic development and based on the mouse phenotype, disrupt neural tube closure (D’Alonzo et al. 2019; Sarkar and Zohn 2012; Zohn et al. 2007). Additionally, our functional analysis demonstrates all the NTD-associated variants identified reduce the activity of HECTD1 related to the etiology of NTDs.

The *HECTD1* sequence variants identified in this study were found in heterozygous NTD cases. Analysis of the gnomAD population database supports the idea that sequence variation in *HECTD1* could cause disease in heterozygotes. *HECTD1* has a high pLI score and a low LOEUF score and is intolerant to missense variation. These metrics are consistent with a haploinsufficient disease gene (Gudmundsson et al. 2022; Lek et al. 2016). Our animal studies also support a haploinsufficient or dominant model for NTDs in mouse models. NTDs are fully penetrant in *Hectd1* mutant mice (D’Alonzo et al. 2019; Sarkar and Zohn 2012; Zohn et al. 2007). Approximately 5% of heterozygous *Hectd1*^*opm*^ mutant embryos exhibit exencephaly with a stop gain mutation at amino acid 144 (L144X), and 20% of heterozygous embryos with disruption of the HECT domain in the *Hectd1*^*Gt(XC266)Byg*^ line show NTDs. Since mutation of the HECT domain creates a dominant-negative protein by preserving the ligase’s interaction with substrates but preventing ubiquitination and degradation of substrates (Huibregtse et al. 1995; Treier et al. 1992), the *Hectd1*^*Gt(XC266)Byg*^ mutation likely has some dominant-negative activity, possibly explaining the higher penetrance of NTDs in this line. One limitation of our study is the lack of available trio sequence data in our cohort, which would help determine the mode of inheritance or if the sequence variants arise de novo. Future studies will be necessary to determine if the NTD-associated *HECTD1* variants identified in this study contribute to NTDs by dominant or haploinsufficient mechanisms.

Genetic analysis of human NTDs indicates oligogenic inheritance with environmental influences (Caiaffa et al. 2023; Chen et al. 2018c; Copp and Greene 2010; Ishida et al. 2018). HECTD1 is a ubiquitin ligase that regulates many pathways required for neural tube closure. Thus, it is also possible that sequence variation in *HECTD1* would interact with additional variants in these pathways to disrupt neural tube closure or contribute to human disease. HECTD1 plays a role in several signal transduction pathways essential for embryonic development, including Notch, Wnt, Retinoic acid**,** and estrogen receptor signaling (Chen and Greenwald 2014; Li et al. 2015; Oikonomaki et al. 2017; Sugrue et al. 2019; Tran et al. 2013)**.** HECTD1 regulates cell migration, focal adhesion assembly, and EMT (Duhamel et al. 2018; Li et al. 2013; Shen et al. 2017; Wang et al. 2020). HECTD1 could broadly influences development by regulating the STRIPAK complex, chromatin remodeling, cholesterol export, and protein translation (Aleidi et al. 2015, 2018; Beard et al. 2016; Lampersberger et al. 2023; Li et al. 2015; Lu et al. 2022; Lv et al. 2021). HECTD1 influences proliferation, apoptosis, autophagy, DNA damage repair, and mitochondrial function (Beard et al. 2016; Bennett et al. 2020; Duhamel et al. 2018; Liao et al. 2023; Lu et al. 2022; Salas et al. 2023; Segref et al. 2014; Uemoto et al. 2022; Vaughan et al. 2022). Intriguingly, the Chinese NTD cohort utilized in this study was collected in a region where folate deficiency is well documented (Liu et al. 2016; Meng et al. 2015). It would be intriguing to investigate whether the NTD-associated variants identified in our study reduce the activities of other HECTD1-regulated pathways. Additionally, we could explore if *HECTD1* variants interact with genetic variation or folate deficiency to cause NTDs.

In this study, *HECTD1*-associated sequence variants were detected in both closed and open cranial and caudal NTDs. However, mutation of *Hectd1* in mice results exclusively in the exencephaly/anencephaly (D’Alonzo et al. 2019; Sarkar and Zohn 2012; Zohn et al. 2007). While most mouse models of NTDs cause exencephaly (Juriloff and Harris 2000), it is also possible that additional variants modify *HECTD1*-associated phenotypes in humans, leading to different NTD subphenotypes. For instance, analysis of rare variants in an NTD cohort suggests that variants in different pathways are linked to distinct types of NTDs (Zou et al. 2020). Among these is an association of craniorachischisis and encephalocele with retinoid signaling, a pathway also regulated by HECTD1 (Sugrue et al. 2019). Moreover, in mouse studies, the penetrance, severity, and type of NTDs are known to be influenced by the genetic background (Wolujewicz et al. 2021). In studies describing exencephaly in *Hectd1* mutant lines, mice were maintained on a C3H/HeJ, 129 or mixed 129:C57BI/6 genetic background (D’Alonzo et al. 2019; Sarkar and Zohn 2012; Zohn et al. 2007), but NTDs are only partially penetrant when the *Hectd1*^*opm*^ allele is crossed onto the C57BI/6 genetic background (unpublished observation). Thus, in mice, genetic modifiers can alter the phenotype of NTDs with a mutation of *HECTD1*, possibly leading to closed rather than open NTDs.

In conclusion, we detected five case-specific rare missense variants in the *HECTD1* gene in a Chinese NTD cohort. All were functionally validated in assays demonstrating reduced protein expression of one variant and reduced function of all variants in an eHSP90 secretion assay, previously linked to the mechanism of NTD formation in mouse models. Our results provide the first report of sequence variation in the *HECTD1* gene potentially contributing to the etiology of human NTDs.

### Supplementary Information

Below is the link to the electronic supplementary material.

Supplemental Table 1 Missense and loss of function tolerance scores for NTD candidate genes. The list was compiled from (Harris and Juriloff 2007, 2010; Ishida et al. 2018) showing Gene symbol and name. If the gene is associated with a human disease, the disease is listed along with the MIM number of that disease; “none” indicates that a human disease is not yet associated with the gene. Missense Z score, pLI, and LOEUF scores from gnomAD v4.0.0 are indicated unless indicated by * for v2.1.1 or not reported (NR). The data was sorted by missense Z score, with the highest score on the top. The data for HECTD1 is highlighted in yellow. HECTD1 has the fifth-highest missense Z score on the list. (XLSX 40 KB)

## Data Availability

The datasets generated during and analyzed in this study are available from the corresponding author upon reasonable request.
